# Embedded Research Fellows: Charting New Paths for Impact: Comment on "Early Career Outcomes of Embedded Research Fellows: An Analysis of the Health System Impact Fellowship Program"

**DOI:** 10.34172/ijhpm.9024

**Published:** 2025-09-30

**Authors:** Patrick Feng

**Affiliations:** Dalla Lana School of Public Health, University of Toronto, Toronto, ON, Canada.

**Keywords:** Embedded Research, Training, Career Development, Learning Health System, Health Services Research, Measuring Impact

## Abstract

With more PhDs working outside of academia, embedded research programs are emerging as one way to broaden the skills of students and bridge the gap between theory and practice. Limited data has been collected on the impact of these programs. The paper by Kasaai et al provides a glimpse into the early career paths of alumni from Canadian Institutes of Health Research’s (CIHR’s) Health Systems Impact (HSI) Fellowship. The results suggest demand for embedded researchers is high and their career prospects are promising. Beyond that, the paper raises several issues that warrant further attention. First is the evolution towards learning health systems (LHSs) and the role embedded researchers might play in this. Second is the potential of embedded researchers to span the worlds of academia and practice. Third is how to measure impact in non-academic research roles. This commentary explores these issues and suggests ways that embedded researcher programs can contribute to each.

## Introduction

 For several decades, the career trajectory of PhD graduates has been shifting away from academia. Where PhDs once set their eyes on academic careers, most now find employment outside of academia. At the same time, demand for data, evaluation, and evidence in health services has risen. Against this backdrop, new training programs are attempting to bridge the gap between academia and practice through embedded research. By providing trainees the opportunity to do research while embedded within a health system organization (HSO), these programs seek to enhance the skills of trainees and expand career opportunities for graduates. These programs hold much promise but, to date, limited data has been collected about their impact.

 The paper by Kasaai et alprovides a glimpse into the early career paths of embedded researchers by providing employment and other career outcome measures from the Health Systems Impact (HSI) Fellowship program in Canada.^1^ Launched in 2017 and funded by the Canadian Institutes of Health Research (CIHR), HSI has provided over 200 fellows the opportunity to work as embedded researchers in over 100 HSOs across Canada. Tracking the career paths of these alumni helps address an important gap in the training literature, namely to what extent do such programs prepare graduates for diverse research careers?

 The data presented in the paper is encouraging. The authors found a 100% employment rate across the 87 alumni they were able to track. Moreover, employment was spread over several sectors: 37% of graduates held academic positions, 37% held senior researcher or scientist positions outside of academia, and 32% were in hybrid roles where they held a professional academic affiliation in addition to their primary employment. These results suggest that demand for those with embedded research experience is high and their career prospects are promising.

 Beyond that, the paper raises several issues that warrant further attention. First is the evolution towards learning health systems (LHSs) and the role embedded researchers might play in this. Second is the potential of embedded researchers to span the worlds of academia and practice. Third is how to measure impact in non-academic research roles. The rest of this commentary explores these issues further.

## Learning Health Systems

 The idea of a LHS has gained widespread recognition.First proposed in the early 2000s, the concept has evolved over time and has been defined by the US Institute of Medicine as:

 “A system in which science, informatics, incentives, and culture are aligned for continuous improvement and innovation, with best practices seamlessly embedded in the delivery process, patients and families as active participants in all elements, and new knowledge is captured as an integral by-product of the delivery experience.”^2^

 A key tenet of the LHS model is using data and evidence to inform decision-making. Related to this is the need to build research capacity within the health system to provide timely evidence.^3^ The notion of using research to accelerate learning is certainly appealing. What is less clear is how to put a LHS model into practice. For example, what types of research are most useful in a LHS? What skills, competencies, and resources are required to do this research? And what organizational and system-level conditions are needed to support the effective use of research?

 The HSI Fellowship program has done an admirable job of increasing research capacity within Canada’s health system, placing over 200 fellows in HSOs across the country in just seven years. Most alumni in the study found employment in research-related positions, giving them the chance to continue applying skills gained through their fellowships. Moreover, 22% of alumni were hired by the HSO in which they were embedded—a strong indication of the value these fellows bring to their host organizations.

 While the data presented show success in finding employment, it says little about how graduates are helping to advance a LHS. For example, are alumni using their skills to inform decision-making? Has learning improved in organizations that hired HSI graduates? Details on what embedded researchers do *in situ* is needed to answer such questions. Further studies on the relationship between embedded research and LHS would be illuminating.

 While the current study does not provide such details, observations from a similar training program—the Ontario Health Teams Impact Fellows—paint a hopeful picture^[1]^. In that program, fellows reported being able to influence decision-making in multiple ways, eg, through research, by participating in working groups, or being invited to leadership meetings. Fellows also reported a shift in attitudes in their host organizations, with co-workers being more likely to see research and evaluation as “essential” rather than “nice to have” following their fellowships. This speaks to the importance of receptor capacity, recognizing that informed decision-making is not just about having evidence, but also having the capacity to make effective use of evidence.^4^ Due to their role as *de facto* knowledge translators, embedded researchers help build receptor capacity within their organizations and further the development of local LHSs.

## Spanning Academia and Practice

 The HSI Fellowship program aims to train early career researchers for diverse roles in the health system. Key to this is an “enriched core competencies framework” meant to broaden the skills of trainees beyond those taught in academic programs.^5^ The framework adds professional skills to traditional research skills and acknowledges the gap between academic research and putting research into practice. This raises the question of how the worlds of academia and healthcare delivery can be spanned, and to what extent programs such as HSI might bridge these worlds.

 To answer this question, it would be helpful to know what employers think of the enriched competencies trainees develop during their fellowship. Skills such as leadership, project management, and networking make intuitive sense, but our understanding of how these skills are enacted in practice is thin^[2]^. The high employment rate of HSI alumni and the sizeable number (63%) who found employment outside of academia suggest that graduates are seen as valuable by employers, but whether this is due to the enriched competencies or other factors is difficult to say.

 Another distinguishing feature of the HSI program is *where* participants carry out their training. As embedded researchers, trainees work in a variety of settings (eg, hospitals, community health centres, policy units) and see firsthand the complexities and challenges of health systems change. Rather than doing academic research that must be translated to knowledge users, trainees learn how to apply their research skills in ways that are informed by and responsive to real world conditions. The result is researchers who have experience working directly with health system stakeholders.

 This is borne out in the data: 37% of HSI alumni held research positions outside of academia and 32% held hybrid roles, with positions in both the health system and academia. Hybrid roles are an example of boundary spanning—building connections that cross organizational, social, and informational silos—and are one possible strategy for bridging the worlds of research and practice^[3]^. Embedded researchers often play a connecting role, not just externally but also within their host organizations, as research tends to cut across silos. Given the range of organizations in which they find employment—both in and out of academia, as well as in academic hospitals and other hybrid organizations—HSI alumni are well-positioned to take on this bridging role.

## Measuring Impact

 As more PhD graduates find jobs outside of academia, there is an opportunity and need to rethink how we measure impact and success. What do these terms mean in the context of embedded research? Tackling this is important not just for career progression but also for demonstrating the value of embedded researchers.

 Research impact can be measured in different ways, each with their own methodological, epistemological, and value assumptions.^8^ For example, the *Canadian Academy of Health Sciences* framework considers impacts under five categories (advancing knowledge, capacity-building, informing decision-making, health impacts, and economic and social benefits) and adopts a “return on investment” perspective.^9^ Building on this, the *Canadian Health Services and Policy Research Alliance* proposes 12 indicators for the “informing decision-making” category.^10^ Both acknowledge the challenge of defining appropriate indicators for measuring impact.

 Assessing embedded research is even more complex. The authors note that embedded research is often oriented towards real-world impact. Given this, we need to move beyond traditional academic metrics and look at (among other things) knowledge uptake and use. For example, presenting to a hospital board is arguably more influential than presenting at an academic conference. Both are examples of knowledge dissemination, but hospital leaders are more likely to be in a position to influence how care is delivered. This is borne out in data from the Ontario Health Teams Impact Fellows program, where fellows prized their relationships with health system leaders/mentors and pointed to these as a key factor in how their research was taken up and used within their host organization. This suggests factors such as leadership and organizational culture play as much of a role when it comes to “impact” as the research itself.

 Perhaps the most direct evidence of impact is the fact that 22% of HSI trainees were hired by their host organization following completion of their fellowship. These organizations saw enough value in their fellows to hire them on a permanent basis. In a resource-constrained environment such as healthcare, this is a sign that embedded researchers have a positive impact. Finding other ways to assess real-world impact should be a priority for the embedded research community.

## Conclusion

 Overall, this paper provides valuable data on the early career paths of embedded researchers in Canada. As the authors note, the literature on PhD careers is sparse and not specific to health services or embedded research; this study addresses an important gap. Moreover, with a 100% employment rate, diverse career paths, and high satisfaction among alumni, the HSI Fellowship program has much of which to be proud. Future studies can add to our understanding by exploring what embedded researchers do in practice, how being embedded in the health system affects their work, and ways to measure real-world impact. Moreover, it would be interesting to explore how a well-funded program such as the HSI Fellowships could be adapted to run in settings with fewer resources, such as rural, northern, and remote communities and lower-income countries. Regardless, it is clear that embedded research fellows are in high demand and well-positioned to contribute to LHSs, boundary spanning, and real-world impact.

## Ethical issues

 Not applicable.

## Conflicts of interest

 Author declares that he has no conflicts of interest.

## Endnotes


^[1]^The Ontario Health Teams Impact Fellows program was launched in 2021 to provide analytic and evaluation support to Ontario Health Teams, a new type of integrated care delivery network announced by the Ontario government in 2019. Modelled after CIHR’s HSI Fellowship program, it placed fellows in host Ontario Health Teams where they worked as embedded researchers for up to a year.
^[2]^CIHR’s enriched core competencies framework underwent a review in 2024. The refreshed framework includes many of competencies identified in the original framework, providing a measure of confidence that those skills originally identified remain valuable.^6^
^[3]^The concept of boundary spanning is well established in the social sciences, with some attention being directed towards embedded research in recent years. See, eg, Vindrola-Padros et al.^7^
